# Ultrasonic disintegration of microalgal biomass and consequent improvement of bioaccessibility/bioavailability in microbial fermentation

**DOI:** 10.1186/1754-6834-6-37

**Published:** 2013-03-18

**Authors:** Byong-Hun Jeon, Jeong-A Choi, Hyun-Chul Kim, Jae-Hoon Hwang, Reda AI Abou-Shanab, Brian A Dempsey, John M Regan, Jung Rae Kim

**Affiliations:** 1 Department of Environmental Engineering, Yonsei University, Wonju, Gangwon-do 220-710, South Korea; 2 Department of Environmental Biotechnology City of Scientific Research and Technology Applications, New Borg El Arab City, Alexandria 21934, Egypt; 3 Department of Civil and Environmental Engineering, Pennsylvania State University, 212 Sackett Building, University Park, PA 16802, USA; 4 School of Chemical and Biomolecular Engineering, Pusan National University, Busan 609-735, South Korea; 5 Current address: Research Institute for Sustainable Environments, Ilshin Environmental Engineering Co., Ltd., Reclean Building 3rd Fl., 692-2 Jangji-dongSongpa-gu, Seoul 138-871, South Korea

**Keywords:** Microalga, Ultrasonication, Cell lysis, Carbohydrate components, Bioenergy

## Abstract

**Background:**

Microalgal biomass contains a high level of carbohydrates which can be biochemically converted to biofuels using state-of-the-art strategies that are almost always needed to employ a robust pretreatment on the biomass for enhanced energy production. In this study, we used an ultrasonic pretreatment to convert microalgal biomass (*Scenedesmus obliquus* YSW15) into feasible feedstock for microbial fermentation to produce ethanol and hydrogen. The effect of sonication condition was quantitatively evaluated with emphases on the characterization of carbohydrate components in microalgal suspension and on subsequent production of fermentative bioenergy.

**Method:**

*Scenedesmus obliquus* YSW15 was isolated from the effluent of a municipal wastewater treatment plant. The sonication durations of 0, 10, 15, and 60 min were examined under different temperatures at a fixed frequency and acoustic power resulted in morphologically different states of microalgal biomass lysis. Fermentation was performed to evaluate the bioenergy production from the non-sonicated and sonicated algal biomasses after pretreatment stage under both mesophilic (35°C) and thermophilic (55°C) conditions.

**Results:**

A 15 min sonication treatment significantly increased the concentration of dissolved carbohydrates (0.12 g g^-1^), which resulted in an increase of hydrogen/ethanol production through microbial fermentation. The bioconvertibility of microalgal biomass sonicated for 15 min or longer was comparable to starch as a control, indicating a high feasibility of using microalgae for fermentative bioenergy production. Increasing the sonication duration resulted in increases in both algal surface hydrophilicity and electrostatic repulsion among algal debris dispersed in aqueous solution. Scanning electron microscope images supported that ruptured algal cell allowed fermentative bacteria to access the inner space of the cell, evidencing an enhanced bioaccessibility. Sonication for 15 min was the best for fermentative bioenergy (hydrogen/ethanol) production from microalga, and the productivity was relatively higher for thermophilic (55°C) than mesophilic (35°C) condition.

**Conclusion:**

These results demonstrate that more bioavailable carbohydrate components are produced through the ultrasonic degradation of microalgal biomass, and thus the process can provide a high quality source for fermentative bioenergy production.

## Background

There has been an increasing interest in use of the renewable and sustainable biomass, namely the third generation feedstock for bioenergy production. Microalgae have gained considerable attention as an alternative biofuel feedstock [1,2] as recent discoveries indicate that most algal biomass is exceedingly rich in carbohydrate and oil [3], which can be converted to biofuels using existing technology. Especially high levels of biogas and biofuel can be produced using carbohydrate of algal biomass via fermentation process [4]. Glucose in algal biomass is the most important monosaccharide affecting the fermentative ethanol production that is greatly dependent upon the composition of carbohydrate components in organic substrates. Bioenergy production from biomass generally requires three sequential processes, i.e., hydrolysis, acidification, and bioenergy generation. Numerous studies using algal biomass have reported that hydrolysis is often the rate-limiting step due to rigid cell walls and cytoplasmic membranes that inhibit or delay subsequent biodegradation in the fermentation processes.

Several methods for algal cell disruption have been evaluated including ultrasonication, bead beating, microwave (at 100°C), osmotic shock (with NaCl) and autoclaving (at 121°C) with varied results [5,6]. Sonication has the advantage of being able to disrupt the cells at relatively low temperatures when compared to microwave and autoclave. In addition, sonication does not require the addition of beads or chemicals, thus decreasing processing cost. Ultrasonication has been commonly used for cell lysis and homogenization, and could be an effective treatment for breaking up the rigid cell envelopes of microalgae [7]. During ultrasonication, sonic waves are transmitted to the microalgal culture. The waves create a series of microbubble cavitations which imparted kinetic energy into the surface of the cells and eventually ruptured the cell walls facilitating the release of carbohydrates and lipids from the cell into the exocellular medium [8]. Acoustic streaming is the other mechanism for using ultrasonication that facilitates the mixing of solution. Such homogenization of algal suspension can improve enzymatic and/or bacterial access to substrates and therefore facilitates the subsequent fermentation process [9,10].

Despite the wide use of algal biomass as a feedstock to produce bioenergy, there are only a few studies that quantitatively determine the compositional distribution of carbohydrate components which affects the productivity of fermentative bioenergy. We previously investigated the feasibility of using ultrasonication as a pretreatment prior to bacterial fermentation of microalgal biomass. Ultrasonication resulted in physical disintegration of microalga cell walls and consequently enhanced fermentative production of hydrogen and ethanol [4]. However, the earlier study did not determine the qualitative and quantitative characteristics of the lysed biochemicals. The main objective of this study therefore was to quantitatively evaluate the performance of sonication on microbial fermentation process and to systematically characterize the biochemical compositions and properties of microalgal biomass before and after the sonication pretreatment in the following ways: (1) investigate the composition of carbohydrate components in both dissolved and solid phases; (2) determine the abiotic conversion of total carbohydrates to the dissolved phase through sonication; (3) measure both surface hydrophobicity and electrical stability of micro-algal cell; and (4) evaluate bioenergy productivity via microbial fermentation of microalgal biomass (compared with soluble starch) under different thermal conditions.

## Results and discussion

### Composition and bioavailability of carbohydrate components from algal biomass

Microalgal suspensions were exposed to four different sonication durations of 0 (non- sonication), 10 (short-term treatment), 15 and 60 min (long-term treatment) at 45°C. No change was found in the concentration of dissolved carbohydrates after 10 min sonication compared to non-sonication (data not shown). A 15 min sonication treatment increased the dissolved fraction of total carbohydrates from 3% to 32%. Further increase of sonication up to 60 min resulted in an insignificant increase of the dissolved fraction, accounting for <1% of total carbohydrates. This result implies that effective algal cell lysis had occurred within 15 min by sonication, resulting in rupture of the cell walls and intracellular materials release to the medium. Ultrasonication of microbes can result in much more hydroxyl groups of carbohydrates and/or lipids on the inner and outer cell surfaces due to extensive cell disintegration and lysis [11]. The hydrophilic nature of saccharide-like substances can also make it possible to increase the solubility of organic materials in culture broth because of the electro-negativity of oxygen atoms in hydroxyl ions [12].

Table 1 shows that total carbohydrates accounted for 37% of the nonsonicated algal biomass. Microalgae such as *Chlorella*, *Chlamydomonas*, *Dunaliella*, *Scendesmus*, and *Tetraselmis* have been shown to accumulate a large amount of carbohydrate (>40% of the dry weight) [13]. Cell wall of the green algae such as *Scenedesmus, Chlorella*, *Monoraphidium*, and *Ankistrodesmus* contains 24–74% of neutral sugars, 1–24% uronic acid, 2–16% proteins, and 0–15% glucosamine [14]. The major fractions of sugars are either mannose/glucose or rhamnose/galactose [14]. Glucose and mannose were the major constituents among the quantified monosaccharides, and accounted for 66.4% (which decreased to 60.4% by a 60 min sonication) and 21.7% (which increased to 25.6% after the sonication) of total monomeric sugars, respectively. The increased portion of mannose might be derived from glucose due to sonication [15,16]. Sonication also resulted in small changes in the concentrations of galactose and glucosamine as minor constituents.

**Table 1:** Effect of sonication treatment on composition and bioavailability of total carbohydrate components

**Sonication time, min**	**0**		**15**		**60**	
	**Non-sonicated**	**Fermented**	**Sonicated**	**Sonicated/fermented**	**Sonicated**	**Sonicated/fermented**
Total carbohydrate, g g^-1^	0.37 (0.01)^a^	0.22	0.37 (0.12)	0.08	0.39 (0.13)	0.09
Glucose (%)	66.38	31.90	63.67	8.03	60.40	7.62
Mannose (%)	21.76	18.81	24.33	2.78	25.60	2.67
Galactose (%)	5.94	5.68	8.66	8.04	10.22	10.12
Glucosamine (%)	5.92	3.06	3.34	2.78	3.78	2.66

^a^ The values in the parentheses show the concentrations of dissolved carbohydrates (g-carbohydrate g^-1^-biomass).

Sonication pretreatment of algal biomass was conducted at 45°C for 15 or 60 min. Both non-sonicated and sonicated algal biomass were mixed with an equivalent volume of fermenting bacteria and subsequently fermented at 35°C for 23 days. Other common monomeric dissolved carbohydrates (such as fucose, rhamnose, and galactosamine) were not detected.

Long-term sonication resulted in large increases in dissolved carbohydrates and in fermentative utilization of the carbohydrates. Table 1 shows that dissolved carbohydrates increased from 1% of biomass in non-sonicated microalga to 12% of biomass after 15 min sonication. A 15 min sonication pretreatment also significantly increased total carbohydrates consumption during the following fermentation stage. The amount of total carbohydrates sharply decreased to 89% for the first 16 days of fermentation, while extremely small amounts of residual carbohydrates consistently decreased in the fermentor up to 23 days. Fermentation of fresh algal biomass at 35°C for 23 days resulted in a decrease in total carbohydrates from 0.37 g-carbohydrate g^-1^-biomass to 0.22 g-carbohydrate g^-1^-biomass, while the fermentation after 15 min sonication significantly reduced the total carbohydrates to 0.08 g-carbohydrate g^-1^-biomass. The improved utilization of carbohydrates by sonication was attributed to both increased dissolved carbohydrate concentrations and increased microbial access to carbohydrates was available on the ruptured cell walls.

### Electrical stability and functional property of microalgal cell

**Figure 1:**
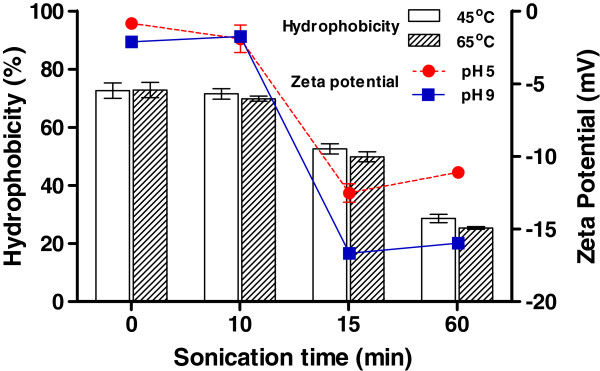
**The cell surface hydrophobicity (left y-axis) and zeta potential (right y-axis) of algal biomass as a function of sonication duration.** Sonication of algal biomass was conducted at two different temperatures of 45°C and 65°C for up to 60 min. The pH of non-sonicated and sonicated algal biomass suspensions was adjusted to either 5 or 9 for zeta potential measurement. Three independent biological replicates were used for the measurements. Error bars indicate standard deviation values from the average.

Several previous studies reported that hydrophobic aggregation decreased bioaccessibility of fermenting bacteria to substrate [17,18]. The surface charge represented by zeta potential is important for a better understanding on the nature of the particle stability [19], and it has also been well-known that colloidal particles with higher absolute values of the zeta potential tend to be less aggregated due to high electrical repulsion among the particles [20,21]. The pH of the mixture of microalgal biomass and inoculum (fermenting microbes) ranged from 5.7 to 6.8 at the beginning of fermentation, and the final pH values after the 23 days of fermentation were nearly identical (5.5 ± 0.2) in all sets of tested samples. Figure 1 shows that the zeta potential of algal biomass was slightly negative at pH 5, became more negative as the pH increased to 9, and was sharply decreased by a 15 min sonication regardless of the initial pH values. Our result indicates that algal cell became more electrostatically stable in aqueous solution at pH 5 and 9 as a result of sonication treatment for 15 min or longer due to exposed functional group as well as the release of negatively charged organic constituents upon cell lysis. This result also coincided with a significant increase of the dissolved carbohydrate fraction in the suspension upon sonication [12].

Sonication treatment for up to 10 min did not significantly changed the algal surface hydrophobicity accounting for 75 ± 3%, but which was decreased to 54 ± 2% or 28 ± 3% by a 15 min or 60 min sonication, respectively, at 45°C. It should be noted that almost identical hydrophobicity was observed in sonication of algal biomass at two tested temperatures. The results reveal that algal biomass was significantly hydrophilized by sonication treatment at 45°C or higher for 15 min. Chen et al. (2004) showed that sonication changed the molecular structure of organic matter, especially resulting in decreases in aromaticity, molecular weight, and specific UV absorbance that has been used as an indicator of organic hydrophobicity [22]. These changes to organic properties were consistent with our observation showing a significant decrease in the surface hydrophobicity of microalga, possibly due to hydroxyl radical production resulting in a substantial degradation of organic compounds and thus an increase in the assimilable organic carbon fraction of the total organic carbon pool [23]. The destruction of algal cell structures during sonication pretreatment also released more algal cell fragment to the aqueous phase, which was observed by increased residual turbidity by 24%. The functionality of organic matter is an important factor in determining how efficient heterotrophic bacteria can assimilate the organic substrates. Numerous studies have demonstrated that heterotrophic microorganisms preferably assimilate hydrophilic organic substrates to a much greater extent than hydrophobic organics [24,25].

Our result showed that the sonication decreased hydrophobicity of cell fragments compared to the control, possibly due to a decrease in the number of conjugated bonds in chain structures [22,26]. Review of the literature demonstrates that algal cells are significantly hydrophilized by ultrasonication [27], which was demonstrated with improved availability of algal biomass as a fermentable substrate for the fermenting bacteria in this study. Hydrophilic functional groups of particle surface also contribute to increasing colloidal stability as shown in zeta potential in this study along with an increase of the specific surface area, resulting in improved bacterial accessibility and metabolic activity [17,18]. These previous findings were consistent with our observation showing an increase of carbohydrate consumption in the microbial fermentation of algal biomass after sonication treatment.

### Effects of sonication duration and fermenting temperature on bioenergy production

**Figure 2:**
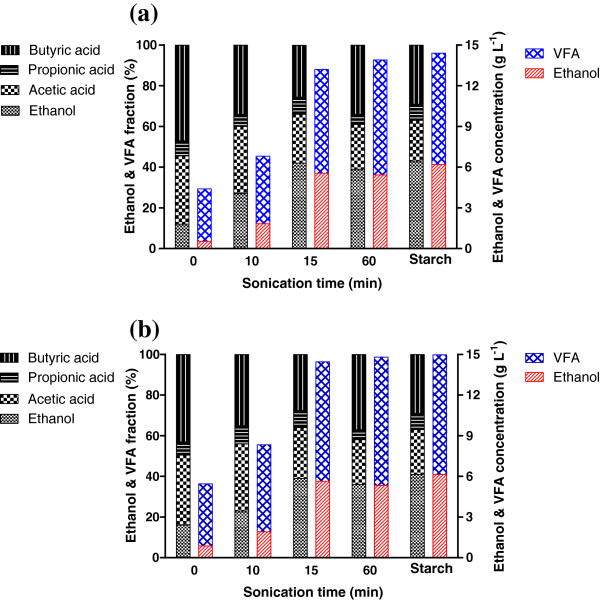
**Effects of sonication duration and fermentation temperature on the production of ethanol and VFA from microbial fermentation of algal biomass.** Either non-sonicated or sonicated (at 45°C) algal biomass was mixed with an equivalent volume of fermentative bacteria, and the mixture was then fermented at a temperature of (**a**) 35°C or (**b**) 55°C for 23 days.

Figure 2 shows the compositional distribution and concentrations of soluble metabolite product (SMP) after the 23 day fermentation of algae biomass under different thermal conditions (35°C and 55°C). The concentrations of ethanol and volatile fatty acid (VFAs) were increased as the sonication duration was increased to 15 min, while the production of those materials was rarely improved even when the sonication duration was increased up to 60 min regardless of the fermentation temperature. Figure 2 also compares algal biomass (both sonicated and non-sonicated) as a substrate with starch as a control for microbial fermentation in terms of the bioenergy productivity. The use of algal biomass sonicated for 15 min or longer was comparable to soluble starch as a feedstock for the production of ethanol/VFAs throughout the 23 day fermentation period. In case of sonication pretreatment for 15 min or longer, butyric acid was the dominant form of VFAs followed by acetic acid and propionic acid. Fermentative biofuel production from organic substances results in incomplete decomposition of substrate into organic acids such as acetate and butyrate. Butyrate is more dominant because of its lower Gibbs free energy (ΔG = −257.1 kJ) compared to acetate (ΔG = −184.2 kJ) and its production involves enzyme activity [28]. This observation was consistent with previously reported work in which the butyrate type fermentation process was employed [4,27,29]. Ethanol has also been reported as the major SMP during anaerobic degradation of saccharide-like substances because monomeric sugars can be utilized easily by heterotrophic microbes (e.g., fermentative bacteria) [30].

**Figure 3:**
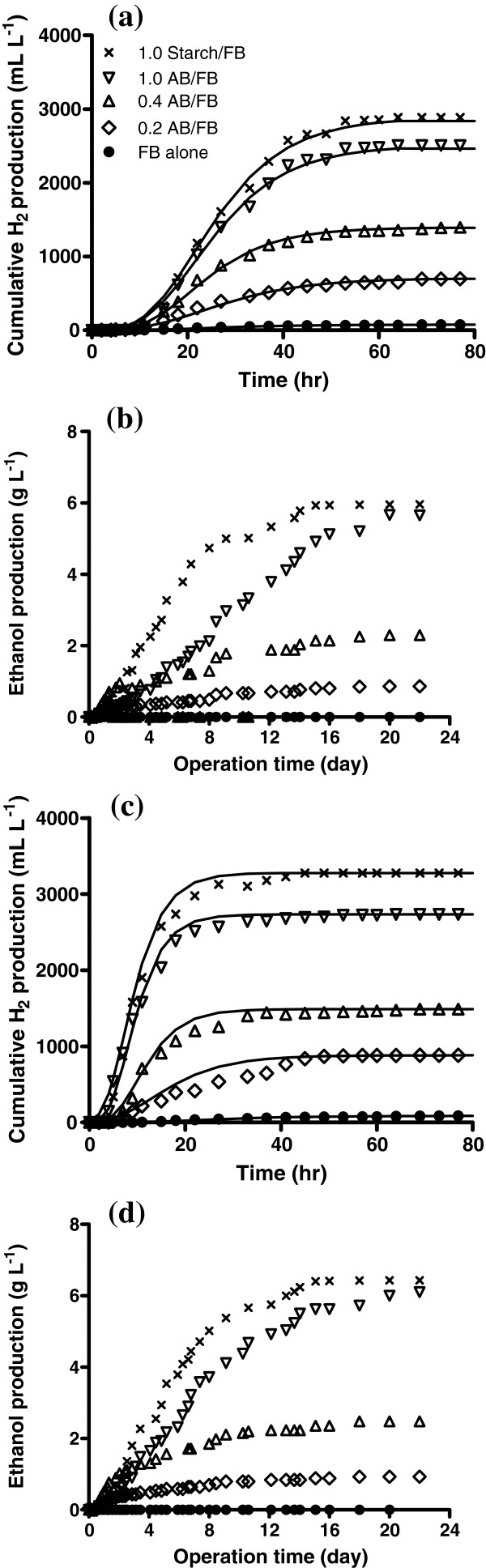
**Effects of the ratio of algal biomass to fermentative bacteria (AB:FB) on the production of hydrogen and ethanol during the 23 day fermentation at a temperature of 35°C (a and b) or 55°C (c and d).** The algal biomass was sonicated at 45°C for 15 min.

The results of microbial fermentations with non-sonicated and sonicated algal biomass are shown in Figure 3 in which cumulative hydrogen and ethanol production are plotted according to time. Production of ethanol resulted in decreased hydrogen production, which was consistent with an earlier report that hydrogen production was decreased when ethanol production was initiated [31]. This was due to a shift of metabolic pathway from butyrate fermentation to ethanol fermentation. The maximum production of hydrogen (H_max_) via microbial fermentation of algal biomass at 55°C was 10% higher than achieved at 35°C, but the fermenting thermal conditions rarely affected the energy conversion efficiency (H_2_ yield). Table 2 shows that the maximum hydrogen production rate (R_max_) was independent of the temperatures between 45°C and 65°C in ultrasonic pretreatment of algal biomass. As the volumetric ratio of algal biomass to fermentative bacteria (AB:FB) was increased from 0.2 to 1.0, the maximum accumulative hydrogen production increased from 0.72 to 2.51 L L^-1^ and from 0.87 to 2.72 L L^-1^ at two different fermenting temperatures of 35°C (mesophilic) and 55°C (thermophilic), respectively. This might be attributed to the improved fermentative bacteria activity at the higher organic loading rate under themophilic conditions. The dissolved carbohydrates concentration increased by sonication was also correlated with the increased H_2_ production, and the remarked production of hydrogen was thus due to improved bioavailability of algal biomass for the fermenting bacteria. The λ (average lag time) values calculated from Gompertz equation are close to those observed in the experiments. The λ value prior to exponential hydrogen production was 11 h under mesophilic conditions, while thermophilic showed much shorter λ (2 to 3 h) and relatively higher R_max_ (up to 0.3 L L^-1^ h^-1^) compared to observed for mesophilic. Our observations were consistent with previously reported work in which anaerobic fermentation of glucose increased the production of hydrogen when operating the fermenter with high organic loading rates under thermophilic conditions [29]. Many factors such as substrates, their concentration, pH and temperature can influence on the fermentative hydrogen production [32,33]. Among them, temperature is a key factor because it can affect the activity of hydrogen producing bacteria (HPB) by influencing the activity of essential enzymes such as hydrogenases for fermentative hydrogen production [34,35].

**Table 2:** Effects of pretreatment temperature and fermentation conditions on hydrogen production from algal biomass sonicated for 15 min at a given frequency and acoustic power

**Sonication temperature (°C)**	**Fermentation temperature (°C)**	**AB/FB ratio (v/v) **^**a**^	**pH**_**i**_^**b**^	**pH**_**f**_^**c**^	**Glucose (g) **^**d**^	**Lag time, λ (hr)**	**H**_**2 **_**yield (mol H**_**2**_ **mol**^**-1 **^**glucose)**	**H**_**max**_^**e **^**(L L**^**-1**^**)**	**R**_**max**_^**f **^**(L L**^**-1**^ **h**^**-1**^**)**	**Note**
45	35	0.2	5.7	5.5	0.09	11	1.6	0.72	0.02	
		0.4	6.4	5.6	0.18	12	1.5	1.39	0.04	
		1.0	6.8	5.4	0.44	12	1.6	2.51	0.16	
		1.0^g^	6.4	5.7	0.50	9	1.9	2.99	0.18	starch
	55	0.2	5.8	5.6	0.09	2	1.5	0.87	0.03	
		0.4	6.4	5.4	0.18	3	1.7	1.48	0.13	
		1.0	6.7	5.3	0.44	2	1.9	2.72	0.27	
		1.0^g^	6.5	5.4	0.50	2	1.9	3.41	0.31	starch
65	35	0.2	5.7	5.4	0.09	12	1.6	0.88	0.03	
		0.4	6.3	5.6	0.18	11	1.8	1.36	0.06	
		1.0	6.8	5.7	0.44	11	1.8	2.94	0.15	
	55	0.2	5.9	5.4	0.09	2	1.7	0.92	0.04	
		0.4	6.2	5.6	0.18	3	1.8	1.33	0.14	
		1.0	6.6	5.6	0.44	3	1.8	2.91	0.30	

^a^ The volumetric ratio of algal biomass (AB) to fermentative bacteria (FB); ^b^ initial pH; ^c^ final pH; ^d^ glucose content in 50 mL of either algal suspension or liquid starch; ^e^ maximum accumulative H_2_ production; ^f^ maximum H_2_ production rate; ^g^ the volumetric ratio of non-sonicated liquid starch to fermentative bacteria (FB).

Ethanol production from algal biomass was increased from 0.9 to 5.6 g L^-1^ with increasing the AB/FB ratio from 0.2 to 1.0 under mesophilic condition. The highest ethanol production among the experimental variations using algal biomass was comparable to the fermentation of equal amount of carbohydrate in starch (see Table 2 and Figure 3b and d). Carbohydrates accounting for 37% of the algal biomass were not only especially beneficial components for heterotrophic bacterial activity, but also a valuable source for fermentative bacteria leading to enhanced energy production.

### SEM images of the sonicated alga

**Figure 4:**
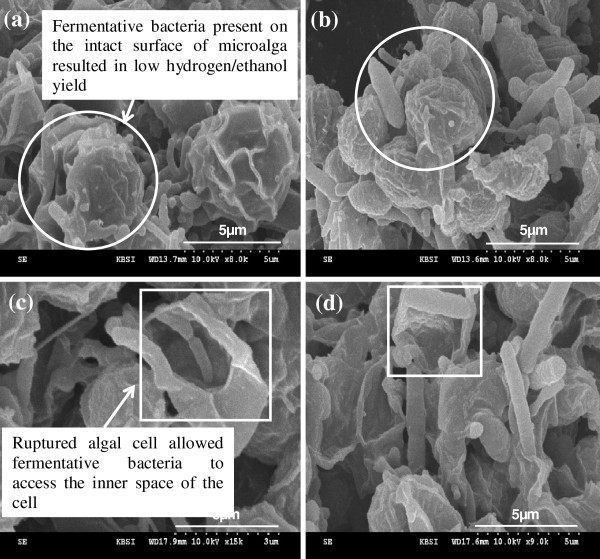
**SEM images of fermentative bacteria along with non-sonicated or sonicated algal cells.** Non-sonicated algal cells (**a** and **b**): (**a**) non- or (**b**) 10 min sonication. Sonicated algal cells (**c** and **d**): (**c**) 15 min or (**d**) 60 min sonication. The circles indicate fermentative bacteria present on the intact surface of algae, while the squares show ruptured algal cells allowed fermentative bacteria to access the inner space of the algal cells, resulting in high hydrogen/ethanol production.

Extensive cell wall damage was observed after a 15 or 60 min sonication which allowed fermentative bacteria to access the inner space of the ruptured algal cell (Figure 4c and d), while an external shape of alga sonicated for 10 min did not look much different from the intact surface of algal biomass on which fermentative bacteria worked (Figure 4a and b). SEM images clearly show greater accessibility of fermentative bacteria to surface-bound carbohydrates of algal debris after sonication ≥15 min compared to control and 10 min sonication. Further some of the nucleus materials in the sonicated alga presumably spread outside the cell due to complete cell lysis, coincided with a significant increase in the dissolved fraction of total carbohydrates after sonication for 15 min or longer (Table 1). Therefore the long-term sonication pretreatment resulted in enhanced bioaccessibility and bioavailability of algal biomass, which led to the increases in carbohydrate consumption and subsequent bioenergy (hydrogen/ethanol) production.

## Conclusions

This study has characterized the carbohydrate components in algal suspension upon sonication that could result in significant changes in the physicochemical properties of algal cell and subsequent enhancement of biodegradability/bioaccessibility for microbial fermentation. Sonication pretreatment for 15 min or longer on algal biomass (*S. obliquus* YSW15) resulted in a large increase in dissolved carbohydrates (composed mainly of glucose), likely due to release from the cell wall and the periplasm. Sonication enhanced fermentative bioenergy (hydrogen/ethanol) production, and resulted in comparable bioenergy production as compared to using soluble starch. Algal surface hydrophobicity was substantially decreased and electrostatic repulsion among algal debris dispersed in aqueous solution was significantly increased by a 15 min sonication treatment, which provided more facile access of the fermentative bacteria to algal biomass for assimilating carbohydrates of the algal cell fragments. A substantial uptake of the carbohydrate by the fermenting bacteria occurred during thermophilic fermentation of algal biomass sonicated for 15 min or longer, coincided with the high bioenergy production (e.g., ethanol 5.6 g L^-1^ and hydrogen 2.5 mL L^-1^). The bioenergy productivity increased with increasing the organic substrate loading rate on the microbial fermentation regardless of the thermal conditions examined in this study. The economic evaluation of using the renewable carbon sources for promoting microbial fermentation concurrent with bioenergy production should be further investigated.

## Methods

### Cultivation of microalga

*Scenedesmus obliquus* YSW15 used in this study was isolated from the effluent of a municipal wastewater treatment plant at Wonju Water Supply and Drainage Center, South Korea [36]. The algal strain was grown in 1 L Erlenmeyer flask containing 0.5 L Bold Basal Medium (BBM) [37]. The culture was incubated on a rotary shaker (SH-804, Seyoung Scientific) at 27°C and 150 rpm under continuous fluorescent illumination with an intensity of 40 μmol m^-2^ s^-1^ for three weeks.

### Ultrasonication

The harvested microalga biomass (34 mg mL^-1^) was placed in a glass bottle and sonicated for 10, 15, or 60 min in a Branson 8510-DTH sonicator (Danbury, Connecticut, USA) at two different temperatures (45 and 65°C). The bath-type sonicator was used in this study due to the localized cavitation produced by horn-type sonicators [38,39]. The sonication was conducted at a constant frequency of 40 kHz and an output power of 2.2 kW for which the ultrasonic energy was applied in continuous (non-pulsed) mode with constant amplitude of 40% with specific supplied energy (Es) 70.6 MJ Kg^-1^.

### Anaerobic inoculums

Seed sludge used in this study was collected from the anaerobic digesters of a municipal wastewater treatment plant (Wonju Water Supply and Drainage Center, Wonju, South Korea). The microbial sludge was heated at 90°C for 30 min to inactivate methanogenic bacteria and to enhance the activity of H_2_-producing bacteria (HPB). The HPB was acclimatized to a synthetic medium in an anaerobic chemostat reactor for 1 month [40]. The medium was prepared daily and stored in a substrate reservoir maintained at 4°C. An anaerobic reactor (2 L of capacity with 1 L working volume) was filled with a mixture of pretreated sludge and synthetic medium, and operated with a hydraulic retention time of 12 h at 35°C.

### Fermentation of microalgae biomass

Microbial fermentation was performed to evaluate the bioenergy production from the non-sonicated and sonicated algal biomasses under both mesophilic (35°C) and thermophilic (55°C) conditions, and which was also compared with soluble starch as a substrate for the fermenting bacteria. The fermentation was carried out in triplicate using 150 mL serum bottles with a working volume of 100 mL. The volume ratio of algae biomass to fermenting bacteria (AB/FB) ranged from 0 (fermenting bacteria alone) to 1.0, and 50 mL fermenting bacteria (dry biomass = 3.4 g L^-1^) was used for each of the experimental variations. The headspace of each bottle was flushed with N_2_ for 15 min to provide an anaerobic environment and then sealed tightly using a butyl rubber stopper and an aluminum crimp. The bottles were placed in a water bath (SH-502S, Seyoung Scientific) kept at a temperature of 35 or 55°C, and gas/liquid samples were periodically collected from the bottles for measurement of SMP throughout the 23 day fermentation period.

### Analytical procedures

The non-sonicated and sonicated algal cells were primarily-fixed using 4% glutaraldehyde for 2 h, secondarily-fixed using 1% OsO_4_ for 1 h, and rinsed with 0.1 M cacodylate buffer (pH 7.4). The resulting samples were then dehydrated with different concentrations of ethanol, sputter-coated with Au-Pd immediately, and examined with a Low-Vacuum Scanning Electron Microscope (LV-SEM, S-3500 N, Hitachi). An ELS-8000 Electrophoretic Light Scattering Spectrophotometer (Ostuka, Japan) was used to determine zeta potential of microalgal cell. Estimation of microalga cell surface hydrophobicity was also performed with the microbial adhesion to hydrocarbon (MATH) test [41].

The concentrations of carbohydrates were determined using an ICS-5000 bio-liquid chromatography (Dionex, USA) with CarboPac PA1 column [42]. The volatile fatty acids (VFAs) were analyzed by a GC-8A Gas Chromatography (Shimadzu, Japan) equipped with a flame ionization detector (FID) and a glass column packed with 10% Reoplex 400. Ethanol was analyzed by a DS 6200 Gas Chromatography (Do-Nam Ins., Korea) equipped with a FID and a DB-624 column (Agilent, USA). Hydrogen was measured using a gas chromatograph (Shimadzu GC-14, Japan) equipped with a thermal conductivity detector and a molecular sieve 5A. The concentrations of total and volatile suspended solids were determined using the Standard Methods [43]. The pH was also measured by a pH meter (Orion 290A).

## Abbreviations

SMP: Soluble Metabolite Product; VFA: Volatile Fatty Acid; Hmax: Maximum Hydrogen Production; Rmax: Hydrogen production rate; HPB: Hydrogen Producing Bacteria; SEM: Scanning Electron Microscope.

## Competing interests

The authors declare that they have no competing interests.

## Authors’ contributions

BHJ, JAC, HCK, JHH and RAA designed and performed research; BHJ, JAC, HCK, JHH, BAD, JMR, JRK, and RAA analyzed data; and BHJ, JAC, HCK, JHH, BAD, JMR, RAA and JRK wrote the manuscript. All authors reviewed and approved the final manuscript.
